# *PT*-Symmetry Breaking and Spin Control in 2D Antiferromagnetic MnSe

**DOI:** 10.1021/acsomega.4c07291

**Published:** 2024-11-13

**Authors:** Hafiz Adil Qayyum, Muhammad Mansha, Shahid Sattar

**Affiliations:** † Department of Physics, College of General Studies, King Fahd University of Petroleum and Minerals, Dhahran 31261, Saudi Arabia; ‡ Interdisciplinary Research Center for Hydrogen Technologies and Carbon Management, King Fahd University of Petroleum and Minerals, Dhahran 31261, Saudi Arabia; ¶ Department of Physics and Electrical Engineering, Linnaeus University, SE-39231 Kalmar, Sweden

## Abstract

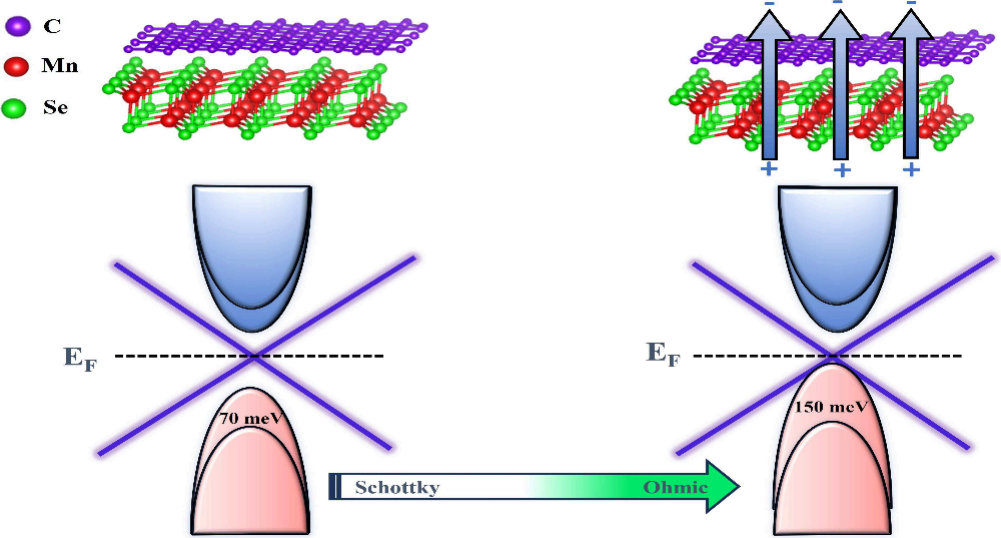

Two-dimensional (2D) materials with intrinsic antiferromagnetic (AFM) order provide a unique avenue to harness both charge and spin degrees of freedom for practical spintronics applications. Here, by using ab initio electronic structure calculations, the interplay of discrete crystal symmetries (such as inversion (*P* ), time-reversal (*T* ), or combined *PT* symmetry) of 2D semiconducting AFM manganese selenide (MnSe) and external electric field along with graphene proximity is investigated. We show that both an external electric field and graphene proximity can independently break otherwise conserved combined *PT* symmetry in 2D MnSe, resulting in large and tunable spin-splittings in both valence and conduction bands, and provide electrical control over a wide energy range. We further propose a current-in-plane electronic device consisting of semiconducting 2D MnSe as a channel material and graphene as a metal contact which preserves not only these features but additionally provides a mechanism to further tune metal–semiconductor contact characteristics such as Schottky barrier height leading to an Ohmic contact. Our results provide a comprehensive insight into the electrical control of the charge and spin degrees of freedom in 2D AFM MnSe.

## Introduction

Magnetic ordering in a semiconductor enables the simultaneous control and utilization of both charge and spin degrees-of-freedom.^1,2^ Unlike ferromagnets (FMs), which are highly sensitive to external magnetic field and generate fringing fields causing unwanted interference between closely packed bits,^3,4^ antiferromagnets (AFMs) are largely insensitive to them owing to their net zero magnetization in the ground-state.^5,6^ Lack of stray fields thus reduces magnetic interactions as well as spontaneous bit flips and permits high-density data storage with minimal power consumption.^5,7^ Moreover, AFMs exhibit ultrafast spin dynamics due to terahertz (THz) scale resonance frequencies compared to FMs operating in the GHz regime,^8^ which makes them ideal for high-frequency spintronics applications.^9,10^ Because of their extraordinary and distinctive features, AFMs are regarded as potential building blocks of next-generation information storage, processing, and neuromorphic computing devices.^11−14^

Discrete crystal symmetries (such as inversion (*P* ), time-reversal (*T* ), or combined *PT* symmetry) play a significant role in gaining a deeper understanding of the electronic structure and properties of two-dimensional (2D) AFM magnets.^15,16^ Semiconducting 2D MnSe, experimentally synthesized by Aapro et al.,^17^ is one such example whose peculiar atomic structure comprised of two FM planes coupled together via AFM ordering ensures net zero magnetization and conservation of combined *PT* symmetry.^18^ The intrinsic nonlinear Hall conductivity in MnSe is theoretically predicted to be orders of magnitude greater than those of existing paradigmatic AFM materials.^19^ Moreover, MnSe multilayers are proposed to exhibit tunable sliding ferroelectricity and magnetoelectric coupling.^20^ However, there are consequences of different external perturbations, together with intrinsic SOC, and lifting of *PT* symmetry on the electronic properties of MnSe. Specifically, the electric field is taken as an external perturbation to induce significant changes in properties of materials. Typical examples include the observation of intrinsic spin Hall effect in nonmagnetic materials,^21−23^ the spin Nernst effect,^24,25^ transformation of antiferromagnetic materials to unipolar or bipolar magnetic semiconductors,^10^ and a full spin polarization in A-type antiferromagnetic materials.^7^ Moreover, to use 2D MnSe in an all-2D current-in-plane device geometry, the contact characteristics (such as Schottky barrier (SB) height and band bending) between the semiconductor and metal electrode are the key parameters that directly influence the functionality and performance of a device.^26,27^ On the other hand, the establishment of an Ohmic contact at the metal–semiconductor interface is highly desirable to achieve efficient charge injection.^28^ Keeping this in mind and motivated by the recent experiments on achieving gate-tunability of various quantities in pristine samples and van der Waals (vdW) heterostructures of 2D magnets,^29−32^ we performed electronic structure calculations based on density functional theory to investigate 2D MnSe under an applied external electric field (*Ê*). Due to the effect of broken *PT* symmetry through *Ê*, we observe large spin-splittings in valence and conduction bands of MnSe which can be gate-tuned over a wide energy range (up to 150 meV for the former). Furthermore, we argue that a vdW heterostructure of MnSe with graphene enhances these splittings to a greater extent. In addition, we propose a current-in-plane electronic device consisting of graphene as a metal contact and semiconducting 2D MnSe as a channel material. By providing an estimate of key quantities such as SB height and band bending, we establish that *Ê* drives this heterostructure from Schottky to an Ohmic contact and provides electrical control of spin splittings and contact characteristics which is highly desirable for all-2D in-plane current devices. These findings provide a detailed understanding of the subject and a potential route to utilize 2D AFM MnSe in next-generation spintronic devices and applications.

## Computational Methods

We performed ab initio electronic structure calculations using the projected augmented wave method as implemented in the Vienna Ab-initio Simulation Package (VASP).^33−35^ The exchange-correlation potential was incorporated using the generalized gradient approximation (GGA) following the Perdew–Burke–Ernzerhof (PBE) scheme.^36^ Interlayer vdW interactions were included using Grimme’s DFT-D3 method.^37^ For the accurate description of strongly correlated Mn d-orbitals, we used the Hubbard *U* correction and set onsite Coulomb (*U*) and exchange (*J*) parameters to the values of 2.3 and 0 eV, respectively, as used in a previous study.^17,18^ The plane-wave cutoff energy was set to 450 eV for pristine MnSe and 550 eV for MnSe/graphene vdW heterostructure calculations. For the structural relaxation, a gamma-centered 12 × 12 × 1 k-mesh was employed, whereas for self-consistent calculations, Brillouin zone integration was performed using a dense 16 × 16 × 1 k-mesh. Owing to its significance, spin-polarization was included in the structural relaxation and all subsequent computations. For the magnetocrystalline anisotropy (MCA) calculations, spin–orbit coupling (SOC) was considered non-self-consistently after an accurate initial collinear self-consistent run. For the iterative solution of Kohn–Sham equations, we achieved an energy convergence of 10^–7^ eV and a force convergence of 10^–3^ eV/Å. To avoid the periodic image interactions, a vacuum region of greater than 30 Å was added in the out-of-plane direction. An external electric field, with strength varying from 0.2 to 0.6 V/Å, was applied in the out-of-plane direction to explore its effects on both pristine MnSe and MnSe/Gr heterostructure. The electric field was treated as an external perturbation with its effects being analyzed on the prerelaxed structures, while keeping other parameters fixed in self-consistent calculations. The raw output files of VASP were extracted and analyzed using the VASPKIT code.^38^

## Results and Discussion

**Figure 1:**
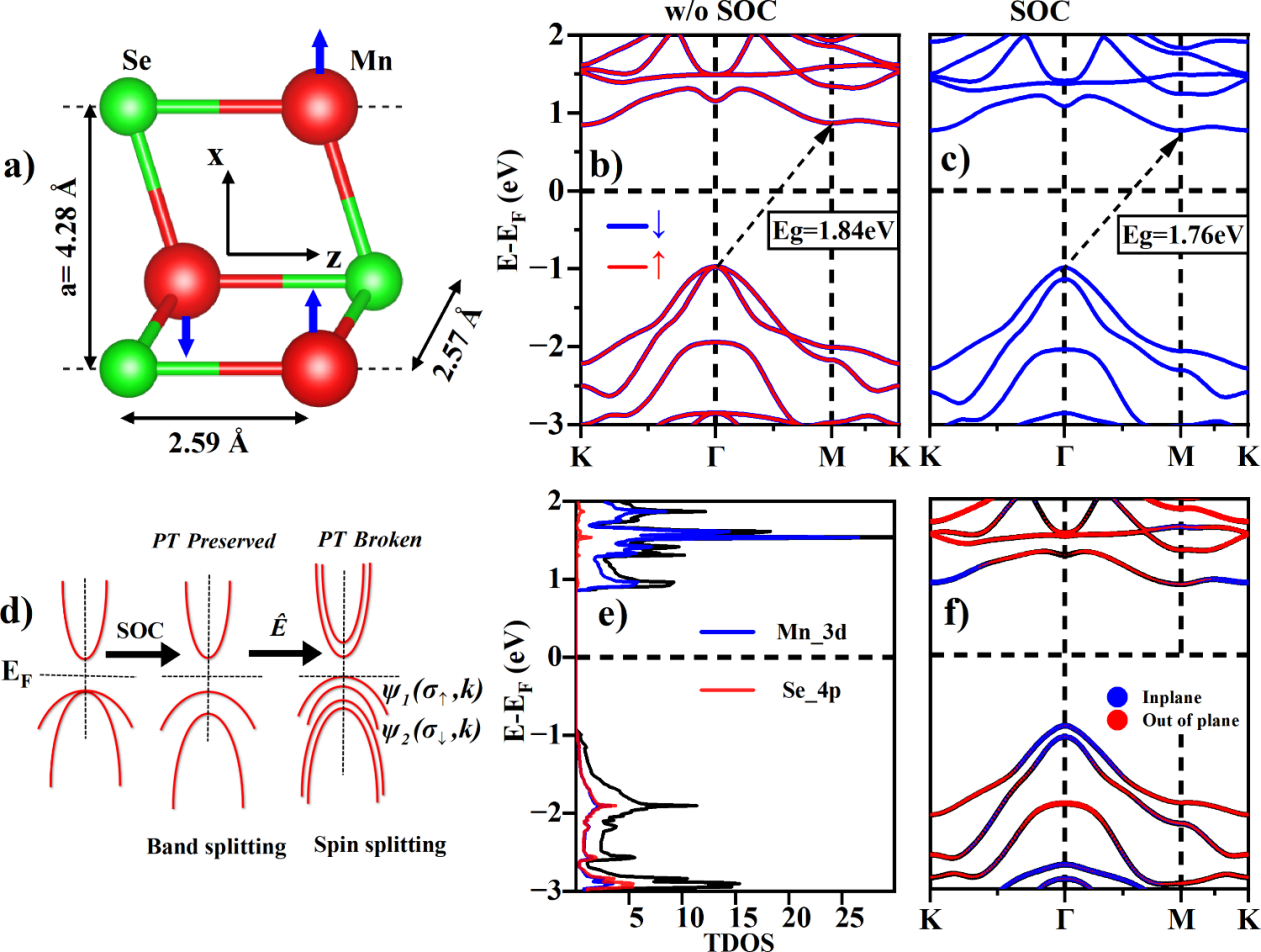
(a) Unit cell of 2D antiferromagnetic MnSe with manganese (selenium) atoms shown in red (green) colors, respectively, along with in-plane and out-of-plane bond lengths. Blue arrows indicate the spin orientation in A-AFM magnetic order. Electronic band structure (b) without SOC and (c) with SOC. (d) Schematic diagram showing the effect of SOC and electric field on band structure of MnSe. (e) Projected density-of-states (DOS) of MnSe. (f) Projected band structure of MnSe showing contribution of in-plane and out of plane orbitals.

Figure 1a shows the side view of the hexagonal 2D AFM MnSe crystal structure belonging to space group *P*3̅*m*1 (No.156) having *C*_3*v*_ point group symmetry. A unit cell of MnSe is comprised of two manganese atoms which are attached from the top and bottom planes to three neighboring Se atoms adopting a trigonal prismatic geometry. The optimized lattice parameter of 4.28 Å with in-plane and out-of-plane Mn–Se bond lengths of 2.57 and 2.59 Å, respectively, is in good agreement with the existing literature.^17,18^ The on-site magnetic moments of upper/lower Mn atoms are +4.36 μ_B_/–4.36 μ_B_, resulting in overall net zero magnetization. To find the most stable magnetic ordering in MnSe, we consider different magnetic configurations, such as ferromagnetic (FM), A-type antiferromagnetic (A-AFM), C-type antiferromagnetic (C-AFM), and G-type antiferromagnetic (G-AFM), as shown in Figure S1. This task is accomplished by constructing a 2 × 2 × 1 supercell of MnSe having 8 Mn atoms. Looking at their energetics, A-AFM is the minimum-energy or ground-state configuration as used in our work. On the other hand, the C-AFM, G-AFM, and FM magnetic configurations are 11, 63, and 200 meV higher in energy, respectively, in comparison to the ground-state configuration of A-AFM. Moreover, the preferable spin orientation in 2D MnSe is also probed via magnetocrystalline anisotropy energy (MAE) calculations. Here, we considered different spin rotations for Mn atoms and compared respective energies obtained under a strict energy convergence criterion (10^–8^ eV). The MAE calculations confirm that an in-plane magnetization in MnSe is more favorable compared to the out-of-plane case, having an energy difference of 0.49 meV/unit cell, in agreement with the existing literature.^18^ To verify thermal stability of A-type antiferromagnetic MnSe, we performed *ab initio* molecular dynamics (MD) simulations at 300 and 400 K for a time-step of 500 fs as shown in Figure S2. With small energy fluctuations and no significant changes in the atomic structure of MnSe, our calculations confirm the thermal stability of MnSe, both at room temperature and at elevated temperatures.

The interplay between local and global symmetries in 2D AFMs is fundamental to understanding their physics. Here, the lattice structure and magnetic ordering are needed to explain this interplay. In 2D MnSe, each Mn atom carries a localized magnetic moment, thus breaking the time-reversal symmetry locally. However, because Mn atoms in neighboring layers align in opposite directions, leading to a net zero magnetization, the combined operation of time-reversal (*T* ) and a spatial translation (*P* ) leaves the system globally time-reversal invariant. This combined symmetry is referred to as *PT* symmetry, a characteristic of 2D MnSe and other experimentally synthesized AFMs like MnBi_2_Te_4_.^39^ An immediate consequence of this symmetry is that the bands are doubly degenerate throughout the Brillouin zone. This feature distinguishes 2D AFM MnSe from ferromagnetic materials, which completely break the time-reversal symmetry. Although *T* and *P* symmetries are individually absent in MnSe, due to static/dynamic *C*_3*v*_ point symmetry and magnetic moments of Mn atoms, the combined *PT* symmetry is still preserved in the monolayer. This combined *PT* symmetry can be expressed as *PT* Ψ(σ_*↑*_,*k*) = Ψ(σ_*↓*_,*k*) where Ψ(σ_*↑*_,*k*)/Ψ(σ_*↓*_,*k*) is the spin up/spin down state configuration along the *k*-path. The MnSe being invariant under *PT* symmetry transformation satisfies the Kramer degeneracy with energy states that are necessarily degenerate, expressed as *E*(σ_*↑*_,*k*) = *E*(σ_*↓*_,*k*). The existence of this *PT* symmetry is reflected in the band structure of MnSe both without and with SOC. Figure 1b showing the band structure of MnSe without SOC depicts an indirect bandgap nature having valence/conduction band maxima/minima at the high-symmetry Γ/M points respectively with a bandgap value of 1.84 eV. The inclusion of SOC lifts the band degeneracy, which can be observed specifically along the high symmetry Γ point in the valence band region with spin–orbit splitting of the order of about 150 meV, and lowers the MnSe bandgap from 1.84 to 1.76 eV as shown in Figure 1c. However, the system is still globally protected by *PT* symmetry (shown in Figure 1d under SOC), which gives rise to the locally dubbed spin states around the high symmetry points with each band under SOC still composed of two nested bands Ψ(σ_*↑*_,*k*) = Ψ(σ_*↓*_,*k*). Figure 1e shows the projected density of states for each element. The valence band region is composed of almost equal hybridization of Mn-d orbitals and Se-p orbitals, while the conduction band is largely composed of Mn-d orbitals with a negligible contribution of Se-p orbitals. To get more insight into the contribution of each orbital around the high symmetry point, we plot the projected band structure of MnSe as shown in Figure 1f. It indicates that the valence band region around the Γ point is mainly formed by in-plane orbitals (p_*x*_ and p_*y*_ for Se and d_*xy*_, d_*x*^2^–*y*^2^_ for Mn), whereas the hybridization of out-of-plane orbitals (p_*z*_ of Se and d_*yz*_, d_*z*^2^_, and d_*xz*_ of Mn) constitute the conduction band region around the M point.

**Figure 2:**
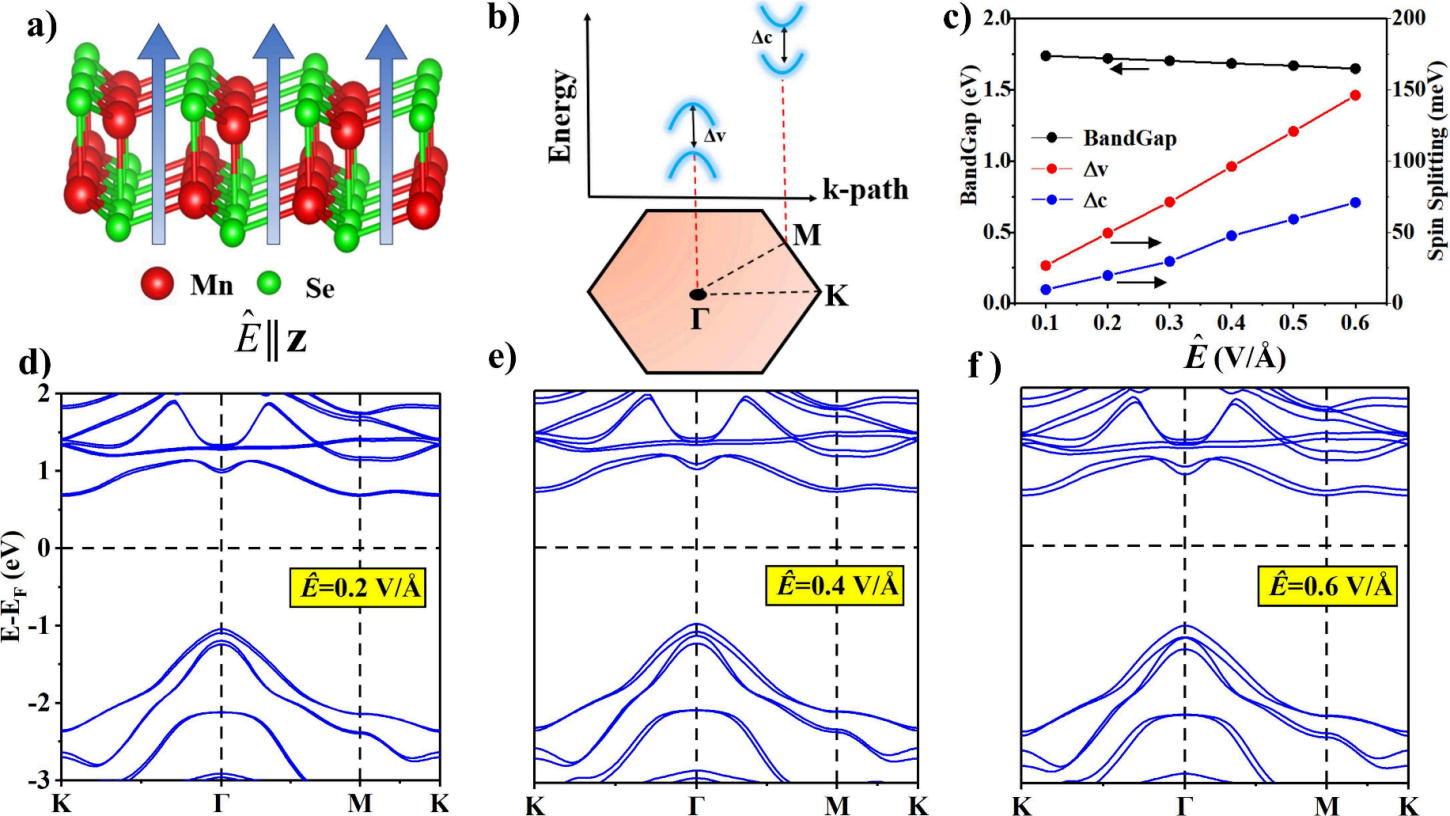
(a) 2D antiferromagnetic MnSe under an applied external electric field (*Ê*) in the out-of-plane direction. (b) Schematic diagram depicting spin splittings in the topmost valence band (Δ*v*)/bottom-most conduction band (Δ*c*) caused by *Ê*. (c) Magnitude of the band gap (in black) and variation in the valence/conduction band spin splittings (Δ*v*/Δ*c*) with respect to applied *Ê* (in red/blue), respectively. (d–f) Electronic band structures under applied external *Ê* of varying magnitudes.

We first apply an external electric field (*Ê*) to pristine 2D MnSe in the out-of-plane direction (*Ê*||*z*) as shown in Figure 2a. Due to a finite potential gradient perpendicular to the 2D plane, *Ê* breaks *PT* symmetry, resulting in lifting the locally dubbed spin degeneracy (Figure 1d under *Ê*) and displaying spin-splitting most notably in the valence band (Δ*v* at the Γ-point) and conduction band (Δ*c* at the *M*-point) as shown in Figure 2b. Furthermore, by gradually increasing *Ê* magnitude, we observe a monotonic increase in spin-split bands with values as large as 150 meV/70 meV for Δ*v*/Δ*c* at a field strength of 0.6 V/Å as shown in Figure 2c. Notice that Δ*v* is much larger than Δ*c* due to p–d orbital hybridization in the valence band compared to the conduction band which is mainly formed by Mn-d orbitals. Moreover, since the valence band maximum at the Γ point and the conduction band minimum at the M point are respectively formed by the hybridization of the in-plane and out-of-plane orbitals, the electric field effect is more profound on the in-plane orbitals. As a result, the spin splitting is larger in the valence band at the Γ-point, while it is comparatively less for the conduction band at the M point. Due to this spin splitting, a small change is also observed in the magnitude of the band gap as depicted in Figure 2c. The electronic band structures at *Ê* values of 0.2–0.6 V/Å are shown in Figure 2d–f. The combined effect of SOC and *Ê* on MnSe can be elaborated in the following way. For each doubly degenerate band of 2D AFM MnSe, SOC merely lifts the bands’ degeneracy at the high-symmetry Γ-point. On top of this, applying *Ê* induces spin-splittings in each band due to nonconservation of *PT* symmetry. Therefore, a finite *Ê* in the out-of-plane direction provides an electrical control of spin-splittings in 2D MnSe to a great extent. In the following, we demonstrate that *Ê* can be further employed to achieve tunable metal–semiconductor contact characteristics.

A 2D current-in-plane electronic device can be built by using semiconducting 2D MnSe as a channel material and graphene as a metal contact. For this purpose, we moved our attention to investigate the vdW heterostructure consisting of MnSe and graphene (MnSe/Gr). Constructing an interface requires both materials to be stacked on top of each other with a minimal possible lattice mismatch. In forming the MnSe/Gr heterostructure, a direct stacking of the graphene unit cell (with a lattice parameter of about 2.47 Å^40^) onto the MnSe unit cell would lead to a significant lattice mismatch. To minimize this, we used  supercell of graphene with a lattice parameter of 4.27 Å, which was stacked onto the unit cell of MnSe. This approach results in an acceptable lattice mismatch of about 0.25% between graphene and MnSe. Figure S3a shows a side view of the optimized MnSe/Gr heterostructure with a calculated interlayer distance of 3.47 Å. We do not observe any significant distortion in either the in-plane or out-of-plane Mn–Se bond lengths within the MnSe/Gr heterostructure. This indicates that the integration of graphene does not disrupt the crystal symmetry of MnSe. Moreover, the interactions between graphene and MnSe are of vdW type, which is confirmed by evaluating the binding energy per C atom (*E*_b_) from the following expression: *E*_b_ = [*E*_MnSe/Gr_ – *E*_MnSe_ – *E*_Gr_]/6, where *E*_MnSe/Gr_ is the total energy of the vdW heterostructure and *E*_MnSe_ and *E*_Gr_ are the energies of isolated 2D MnSe and graphene, respectively. Our computed binding energy of −41 meV for the 2D MnSe/Gr heterostructure is comparable to earlier studied vdW heterostructures (−48 meV for PtSe_2_/Gr^41^ and −31.95 meV for WS_2_/Gr^42^), which confirms that 2D MnSe binds weakly with graphene and the interactions are rather homogeneous.

**Figure 3:**
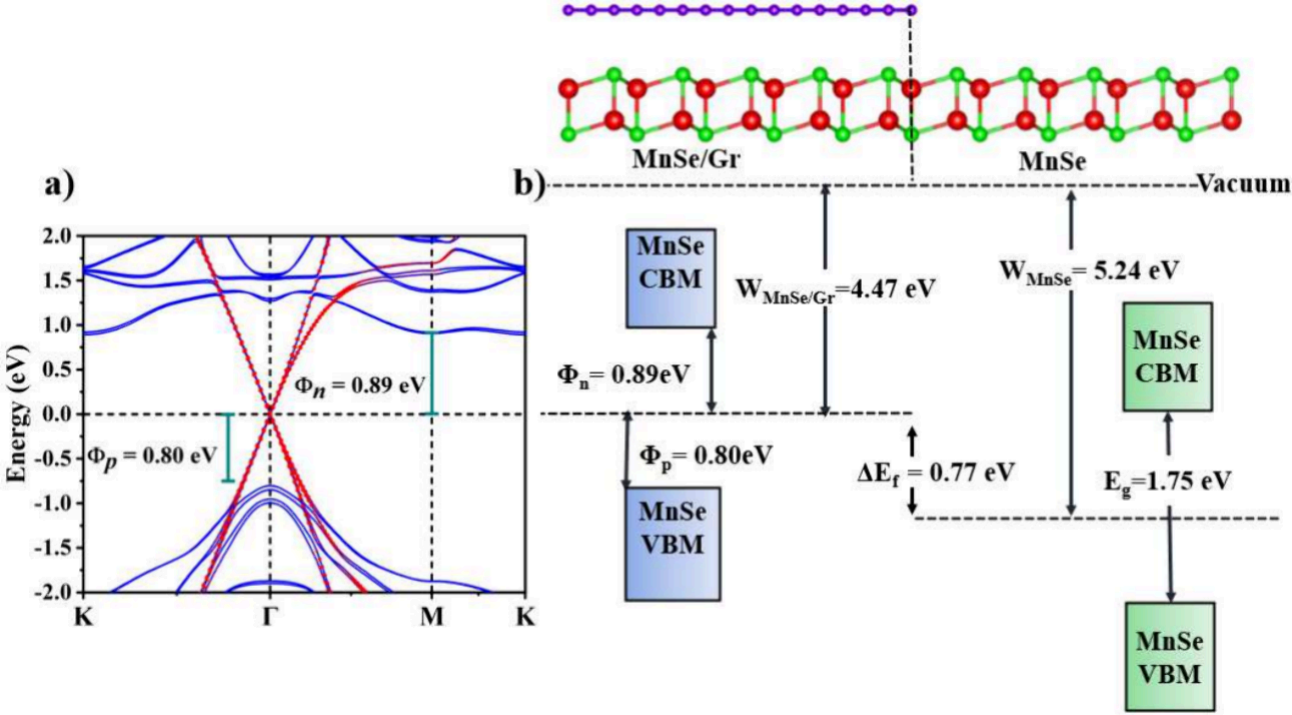
(a) Layer-projected band structures of the MnSe/Gr vdW heterostructure (blue/red colors represent contributions from MnSe and Gr, respectively). (b) Energy-band diagram of MnSe/Gr showing band edges, work function, and estimation of band bending.

Figure 3a shows the layer-projected band structure of the MnSe/Gr heterostructure. Due to no obvious chemical interaction, both MnSe and graphene components preserve their electronic dispersions with bands being merely a superposition of the constituent materials. We observe that graphene, in proximity to 2D MnSe, also breaks *PT* symmetry and thus lifts the spin degeneracy of MnSe (shown in blue) without the need for an external *Ê*. The effect of graphene proximity on the MnSe is further elaborated by considering spin density, the charge density difference, and the electron localization function, as shown in Figure S4. Although the upper and lower Mn atoms are coupled antiferromagnetically in the same manner as in the case of pristine MnSe as revealed by spin density analysis, a small interlayer charge transfer occurs between graphene and MnSe, where the charge depletion occurs around graphene atoms while the Se atoms nearest to graphene attain the charge accumulation. This nonsymmetrical charge distribution induces a delocalization in the electronic density of Se atoms that are close to the graphene as analyzed in the electron localized wave function (ELF) shown in Figure S4d. The existence of this nonuniform charge distribution between graphene and the nearest Se atoms induces a potential difference within the MnSe/Gr interlayer region. To verify this, we calculate the planar-average electrostatic potential as shown in Figure S3c, which reveals a potential drop of about −13.2 eV. This potential difference creates an interlayer built-in electric field, the strength of which can be calculated by considering the gradient of the potential in this region. Our analysis reveals a strong built-in electric field of magnitude 3.78 V/Å between the MnSe and graphene interlayer. Consequently, the stacking of graphene on MnSe results in a significant built-in electric field that effectively induces *PT* symmetry breaking even in the absence of an external electric field. Referring to Figure 3a, the spin splitting induced due to the *PT* symmetry breaking by the graphene proximity is on the order of about 46 meV and can be tuned to larger values using external *Ê* on the MnSe/Gr heterostructure. On the other side, the bandgap opening of approximately 5 meV in graphene was also observed, which is attributed to the presence of the built-in electric field. However, this gap is much smaller than the room temperature thermal fluctuations (25 meV). Therefore, we anticipate that a graphene layer will still effectively behave as a metal and suffice for the need for a source/drain contact material. Furthermore, the appearance of the Dirac bands of graphene at the Γ point in the MnSe/Gr heterostructure is the consequence of the Brillouin zone folding. As illustrated in Figure S3b, the reciprocal space corresponding to the MnSe/Gr heterostructure can be represented in the extended zone scheme, where the high symmetry K point is equivalent to the Γ′ point of the adjacent reciprocal space. Despite vdW interactions, the formation of contact leads to an electrical impediment in terms of band bending when a heterojunction is formed. Such a band bending appears because of the misalignment of the heterostructure work function with that of freestanding materials. Here, we calculate band bending by evaluating the difference between the work functions of pristine 2D MnSe and MnSe/Gr heterostructure using the expression^43^ Δ*E*_f_ = *W*_MnSe/Gr_ – *W*_MnSe_, where *W*_MnSe/Gr_ is the work function of the MnSe/Gr heterostructure and *W*_MnSe_ is the work function of pristine 2D MnSe. For the latter, the work function turns out to be 5.24 eV while, for the MnSe/Gr heterostructure, we observe a significant decrease in work function to a value of 4.47 eV with a calculated band bending of −0.77 eV as shown in the energy-band diagram of Figure 3b. The negative value of the band bending(Δ*E*_f_ < 0) infers that the transport at the contact occurs via electrons flowing from MnSe/Gr to the freestanding MnSe making the channel n-type. As the Schottky barrier (SB) is formed at the MnSe/Gr interface region, it is imperative to provide an estimate of the SB height which can directly be measured in experiments. An SB height can be computed relative to valence/conduction bands (either p- or n-type) by taking the energy difference between their respective band edge and the Fermi energy using the expressions^44,45^ ϕ_n_ = *E*_c_ – *E*_F_ and ϕ_p_ = *E*_F_ – *E*_v_, where *E*_c_/*E*_v_ and *E*_F_ are the conduction/valence band edges and Fermi energy, respectively. We note that the MnSe/Gr heterostructure forms a p-type Schottky contact as the valence band is closer to the Fermi energy (ϕ_p_ = 0.80 eV) compared to the conduction band (ϕ_n_ = 0.89 eV) as shown in Figure 3a.

**Figure 4:**
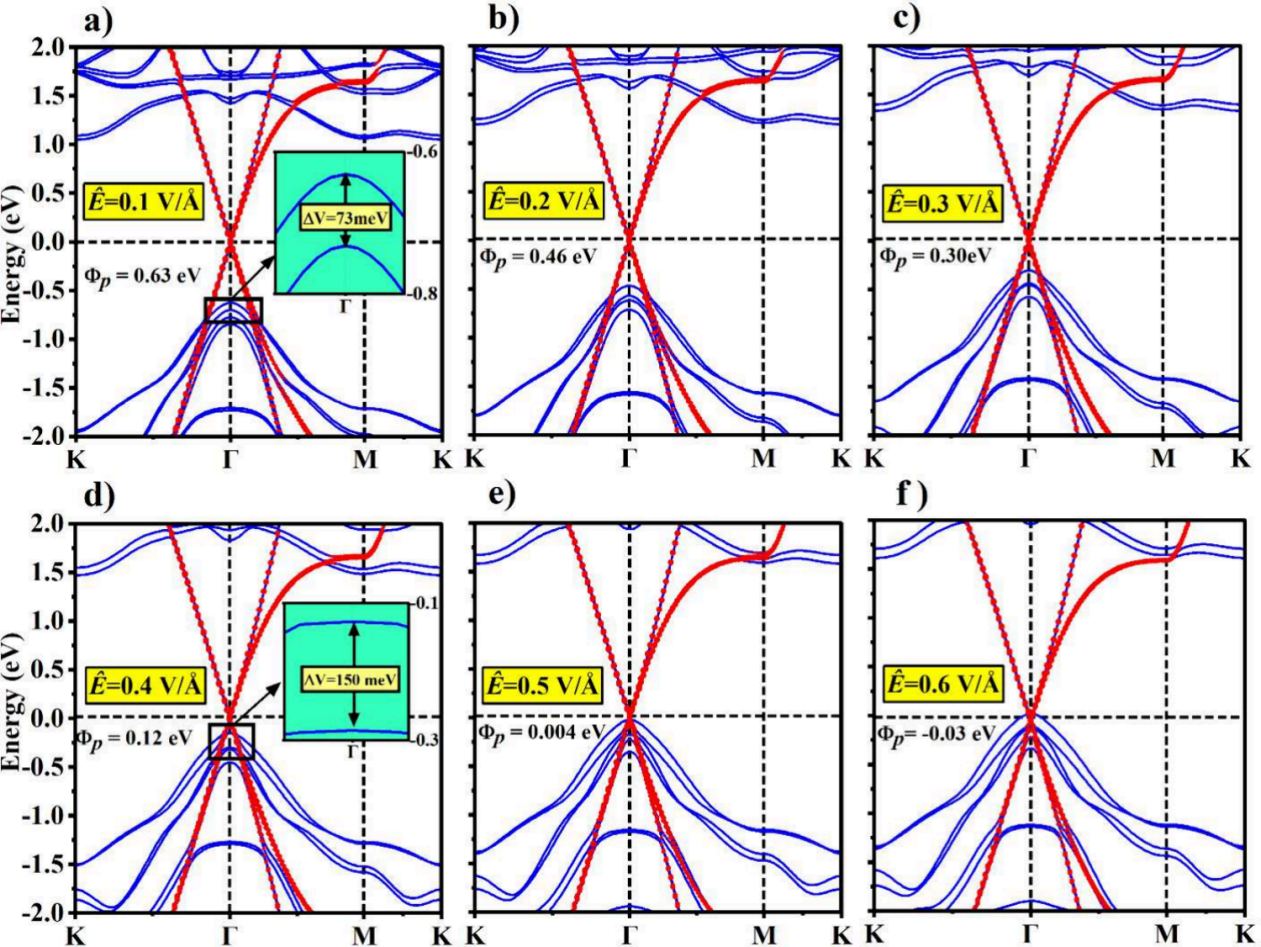
(a–f) The projected electronic band structures of MnSe/Gr under different values of *Ê*. The red and blue symbols denote the contributions from graphene and MnSe, respectively. The insets in (a) and (d) show the valence band splitting in MnSe.

Ideally, the SB height should be zero in a current-in-plane electronic device, implying an Ohmic contact with no opposition to the flow of charge carriers. It is therefore crucial to achieve tunability of the SB height. We achieve this by applying an external *Ê* to the MnSe/Gr heterostructure. Figure 4a–f shows a series of band structures for the MnSe/Gr heterostructure under varying magnitudes of *Ê* directed from MnSe to graphene. The Dirac cone of graphene is still preserved at the Γ point with no significant opening, which confirms graphene enduring behavior as a metal electrode in the heterostructure under increasing *Ê*. However, we notice that an increasing *Ê* induces an upward shift in the valence band of MnSe and brings it close to the Dirac point. This consequently decreases the magnitude of ϕ_p_. In contrast, an upward trend in the conduction band increases the magnitude of ϕ_n_ with increasing *Ê*. This keeps the MnSe/Gr contact as a p-type throughout the electric field variation. Most importantly, in proximity to graphene, which adds up to the applied external field, spin-splittings in MnSe bands are greatly enhanced compared to the pristine case. To quantify this, even at a small *Ê* value of 0.1 V/Å, Δ*v* is about 73 meV (see inset of Figure 4a), which is nearly 3 times larger than the pristine MnSe value. We note that for pristine 2D MnSe, an *Ê* magnitude of 0.6 V/Å is needed to induce Δ*v* on the order of 150 meV. However, this can be accomplished using a significantly lower amplitude of *Ê* (0.4 V/Å) in the MnSe/Gr heterostructure. Therefore, graphene proximity together with *Ê* not only breaks combined *PT* symmetry but also induces large and tunable spin-splitting in 2D MnSe. We highlight the work of Wang et al. in ref (46), demonstrating such gate tunability in CrI_3_/Gr heterostructure and anticipate that it is also viable in this case. Hence, the MnSe/Gr heterostructure offers tremendous potential for the design of efficient current-in-plane electronic devices. We note that numerous theoretical studies suggest the need for high electric field strength to observe novel effects in various materials. For example, topological transitions in phosphorene and transition metal dichalcogenides require an electric field exceeding 0.2 V/Å.^47,48^ Similarly, achieving valley polarization in WSe_2_/CrSnSe needs an immense electric field of about 0.6 V/Å,^49^ while the structural phase transitions in monolayer tellurium are achieved at an electric field greater than 0.7 V/Å.^50^ On the experimental side, ongoing research has made significant progress, with electric field strength exceeding 0.4 V/Å having been achieved through the dual ionic gating method to tune the bandgap of few-layer WSe_2_.^51^ The achievement of a 150 meV spin splitting at a similar electric field strength in our MnSe/Gr system highlights the practical feasibility of our work in experiments.

**Figure 5:**
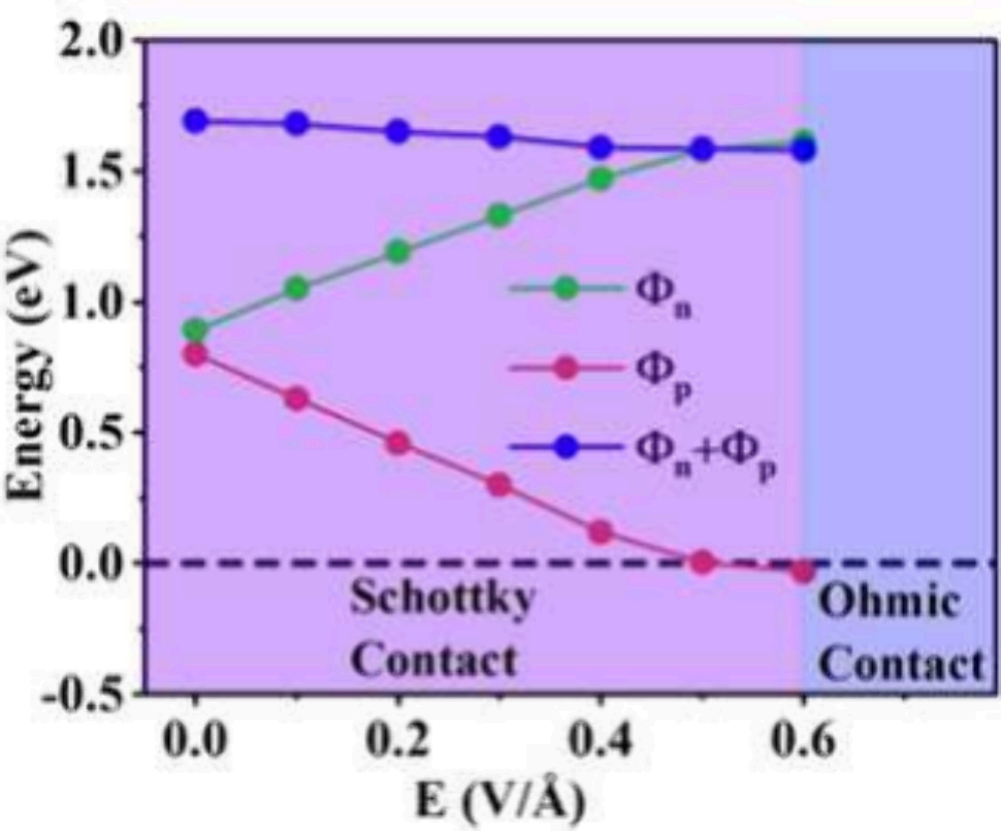
Variations in the SB with an external electric field.

Finally, we provide an estimation of the SB height and transformation from Schottky to an Ohmic contact in MnSe/Gr heterostructure as the function of applied *Ê* in Figure 5. Here, we note that the critical value of *Ê* needed is 0.6 V/Å when the Dirac cone is below the valence band maxima indicating a transition from Schottky to an Ohmic contact. It is also pertinent to mention that the relative positions of valence and conduction bands of MnSe change under *Ê*. Nevertheless, the semiconducting nature of MnSe has been maintained with the bandgap values varying between 1.69 and 1.58 eV (the sum of ϕ_p_ and ϕ_n_).

## Conclusion

In summary, by employing ab initio electronic structure calculations, we investigate the electronic properties of 2D AFM MnSe and MnSe/Gr heterostructure under an applied external electric field. While SOC lifts band degeneracy at the high symmetry Γ-point, both *Ê* and graphene proximity break otherwise conserved *PT* symmetry in MnSe and induce large spin-splittings in the valence and conduction bands. We also note that the splittings are enhanced in the MnSe/Gr case due to the inclusive nature of both mechanisms (*Ê*, and graphene proximity) with broken *PT* symmetry due to graphene proximity as the result of the inequivalent charge distribution between the graphene and the nearby Se atom. To practically utilize these effects, we proposed a current-in-plane electronic device consisting of graphene as a metal contact and semiconducting 2D MnSe as a channel material. By providing an estimate of Schottky barrier height, band bending, and other quantities, we establish that *Ê* drives transformation from a Schottky to an Ohmic contact and provides electrical control of spin splittings and contact characteristics which is highly desirable for all-2D in-plane current devices. These findings provide comprehensive insights and a potential route to utilize 2D AFM MnSe in next-generation spintronics devices and applications by offering control over the spin states even at lower electric field strength.

## Data Availability

The data that supports the findings of this study is available in the manuscript as well as in the Supporting Information file.

## Supporting Information Available

The different magnetic configurations for MnSe, the molecular dynamics (MD) simulation of 2D antiferromagnetic MnSe, crystal structure, the corresponding extended Brillouin zone scheme, and the averaged electrostatic potential associated with the MnSe/Gr heterostructure, and the spin density, charge density difference, and electron localization function (ELF) corresponding to the MnSe/Gr heterostructure (PDF)
