# Supplementary material for: PT-Symmetry Breaking and Spin Control in 2D Antiferromagnetic MnSe
Source: ACS Omega. 2024 Nov 13;9(47):47097–104. doi: 10.1021/acsomega.4c07291 (PMC11603246; doi:10.1021/acsomega.4c07291)
Supplement: Supplementary file 1 — ao4c07291_si_001.pdf [file ao4c07291_si_001.pdf]

$\boldsymbol{PT}$-Symmetry Breaking and Spin Control in 2D Antiferromagnetic MnSe

Hafiz Adil Qayyum [a,b*], Muhammad Mansha[b], Shahid Sattar[c*]

Affiliations:

[a] Department of Physics, College of General Studies, King Fahd University of Petroleum and Minerals, Dhahran 31261, Saudi Arabia.

[b] Interdisciplinary Research Center for Hydrogen Technologies and Carbon Management, King Fahd University of Petroleum and Minerals, Dhahran 31261, Saudi Arabia.

[c] Department of Physics and Electrical Engineering, Linnaeus University, SE-39231 Kalmar, Sweden.

Corresponding Authors email:

hafizadil.qayyum@kfupm.edu.sa; shahid.sattar@lnu.se

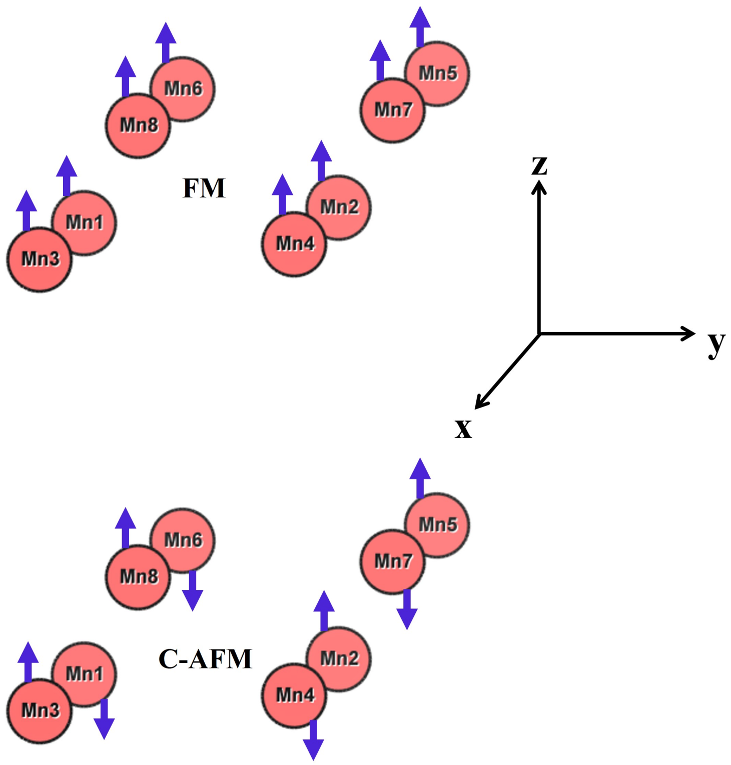

Figure S1. Different magnetic configurations considered to check magnetic ordering in 2D MnSe. Here, Se atoms are removed to improve the visibility of Mn atomic spin configurations.

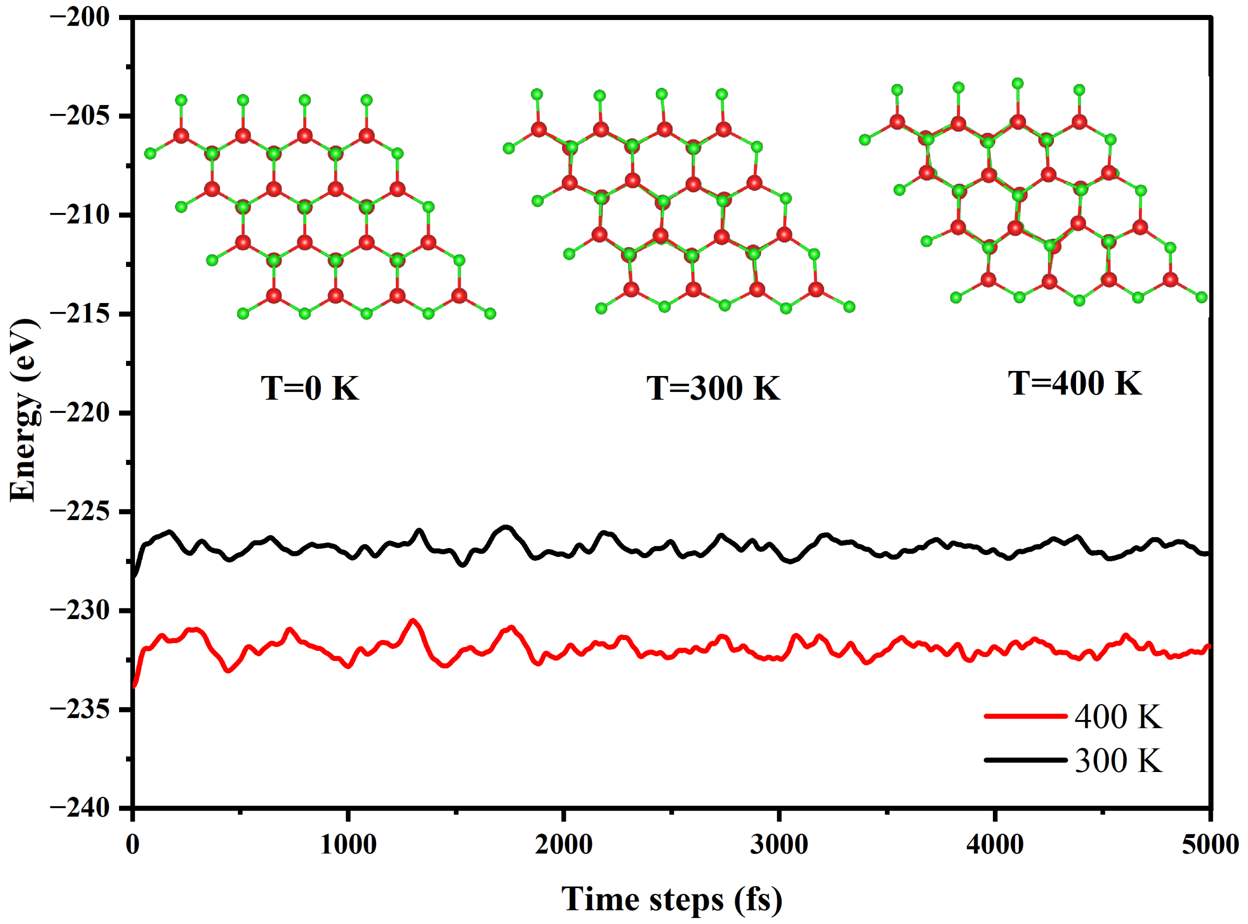

Figure S2. *Ab-initio* molecular dynamic (MD) simulation of 2D antiferromagnetic MnSe at 300 K and 400 K. The inset shows the evolution of the structural change with temperature.

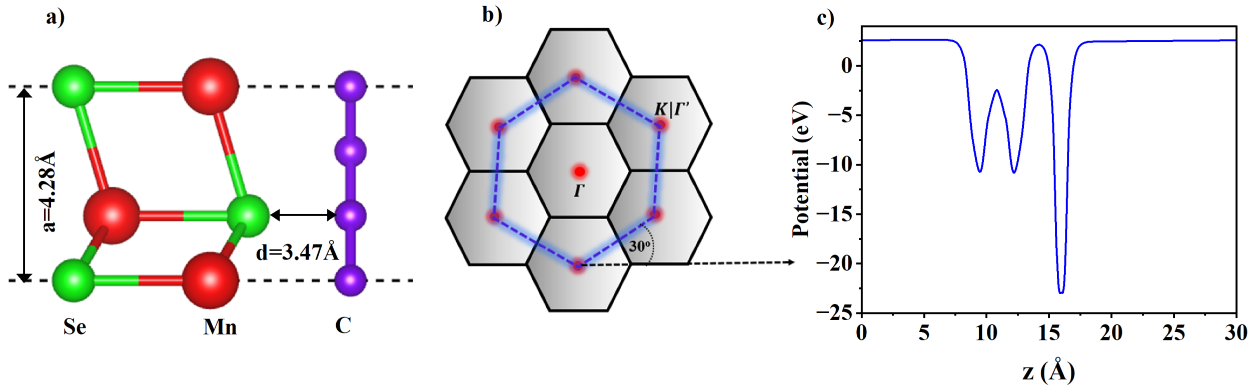

Figure S3. (a) Crystal structure of MnSe/Gr heterostructure (b) the conventional and extended Brillouin zone scheme represented in grey and blue color respectively. (c) Planar-average electrostatic potential for MnSe/Gr heterostructure.

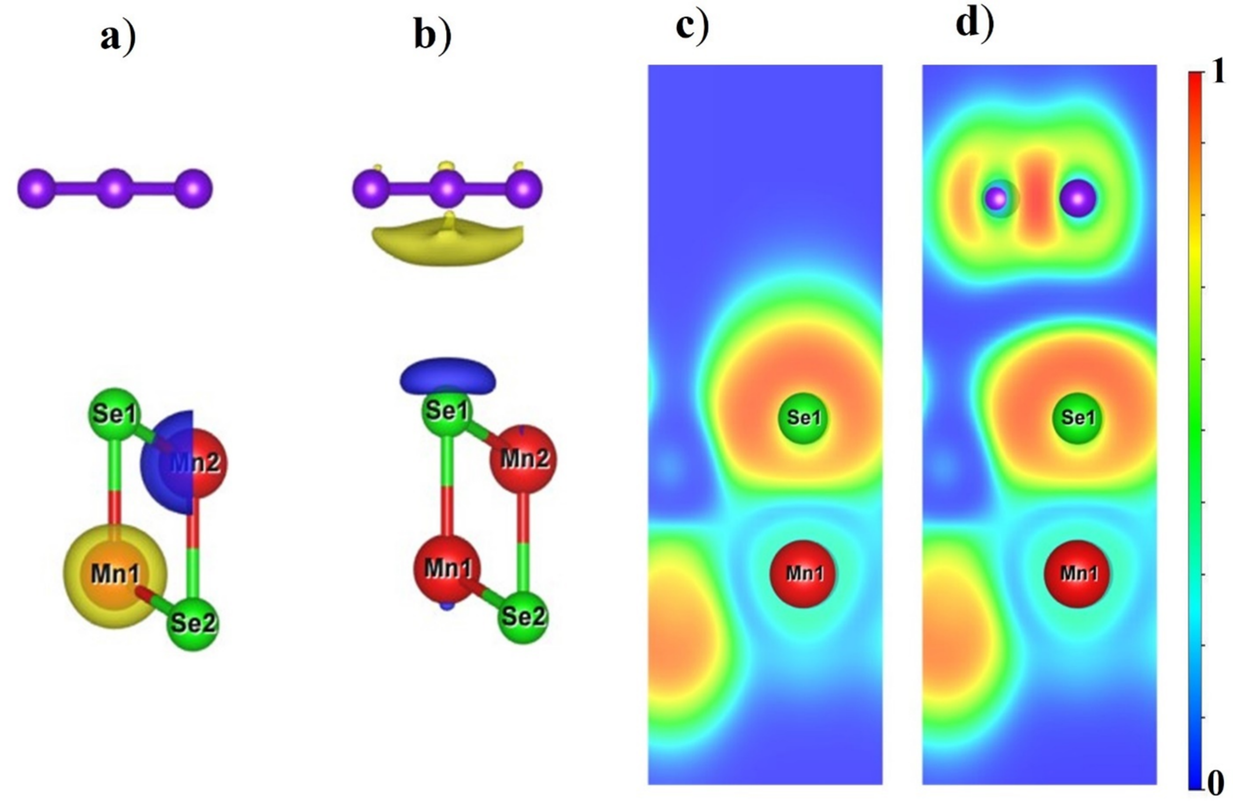

Figure S4. (a) Spin density (isovalue: 0.05 electrons/Bohr^3)) of MnSe/Gr vdW heterostructure.(b) Charge density difference (isovalue: 2×10^{-4}electrons/Bohr^3 of the same. Blue and yellow colors in (a) represents spin-up and spin-down densities, whereas in (b) represents charge accumulation and depletion, respectively.(c,d) Comparison of the electron localization function (ELF) of pristine MnSe and in proximity to graphene, respectively.
